# Olfactory dysfunction and submicroscopic abnormalities: unveiling hidden pathologies

**DOI:** 10.3389/fnmol.2026.1861373

**Published:** 2026-09-30

**Authors:** Katarzyna Bilińska, Magdalena Pasińska, Fernando de Castro

**Affiliations:** 1 Department of Clinical Genetics, Faculty of Medicine, Collegium Medicum in Bydgoszcz, Nicolaus Copernicus University, Bydgoszcz, Poland; 2 Centro de Neurociencias Cajal-CSIC, Consejo Superior de Investigaciones Científicas-CSIC, Madrid, Spain

**Keywords:** anosmia, anosmin-1, copy number variants, Kallmann syndrome, microdeletion, microduplication

## Abstract

Recent advances in high-resolution genomic technologies have revealed that several syndromes are associated with submicroscopic chromosomal deletions and, less frequently, duplications, thereby substantially contributing to the functional annotation of the human genome. Among these structural variants, recurrent microdeletions or microduplications often disrupt dosage-sensitive genes, leading to shared phenotypic outcomes in affected individuals. While features such as congenital heart defects, neurodevelopmental delays, and intellectual disability (ID) have been extensively studied, phenotypes resulting in olfactory dysfunction remain largely underexplored. Here, we review reported cases of copy number variants (CNVs) in patients with isolated or syndromic olfactory impairment and stratify the strength of associations between CNVs and olfactory dysfunction using predefined evidence levels. The available evidence ranges from well-supported *loci* with documented human olfactory phenotypes and mechanistic evidence to hypothesis-generating observations based primarily on isolated cases or biological plausibility. By distinguishing these levels of evidence, we highlight both established dosage-sensitive pathways involved in olfactory development and major limitations of the current literature, including incomplete olfactory phenotyping and the difficulty of interpreting large multigenic CNVs. This framework may facilitate more rigorous genotype-phenotype interpretation in congenital and syndromic olfactory disorders.

## 1 Introduction

Olfactory disorders represent a heterogeneous group of conditions (congenital and acquired) characterized by partial or complete loss of smell and significantly impacting quality of life (Croy et al., 2014). While non-genetic factors (e.g., prenatal or postnatal infections, toxic exposures) may contribute to congenital olfactory dysfunction (Schäfer et al., 2021), the congenital forms may present as isolated congenital anosmia (ICA), with varying degrees of hyposmia, or as part of syndromic disorders, such as Kallmann syndrome (KS), characterized by the coexistence of anosmia and hypogonadotropic hypogonadism (HH; Karstensen and Tommerup, 2012). Increasing evidence suggests that genetic alterations play a key role in congenital forms (Kamarck et al., 2024).

In genetics, copy number variants (CNVs) have emerged as an underappreciated mechanism that affects the development and function of the olfactory system (Shaikh, 2017). Advances in molecular cytogenetics and high-resolution genomic techniques have facilitated the detection of submicroscopic chromosomal aberrations, including microdeletions and microduplications, in patients with olfactory deficits. These structural variants can, for example, disrupt dosage-sensitive genes involved in the development of olfactory structures, thereby contributing to abnormal smell perception (Kim et al., 2023). Although the number of reported cases remains limited and many associations are based on single observations, accumulating evidence indicates that CNVs represent an important source of genetic heterogeneity in congenital olfactory dysfunction. Recognizing these structural variants not only expands the spectrum of genetic mechanisms underlying smell disorders but may also provide insights into broader developmental pathways, guiding future diagnostic strategies. Other potential genetic mechanisms contributing to olfactory dysfunction will also be discussed below.

Our present work aims to review the contribution of CNVs to congenital and syndromic olfactory disorders and to summarize genomic regions and candidate genes associated with these phenotypes. We conducted a structured review of publicly available genomic databases and the literature to identify CNVs reported in individuals with anosmia, hyposmia, or structural abnormalities of the olfactory system (Supplementary Table S1). Importantly, because the available evidence ranges from validated olfactory phenotypes to isolated database observations to indirect biological support, we additionally stratified CNV-olfactory dysfunction associations by predefined evidence levels (Table 1). This approach allows well-supported associations to be distinguished from provisional or hypothesis-generating *loci* (Figure 1). Beyond its interest to biomedical researchers, this review is intended as a practical reference for pediatricians, clinical geneticists, cytogeneticists, and molecular geneticists. It highlights both established CNV-associated olfactory phenotypes and genomic regions warranting further investigation.

**Figure 1:**
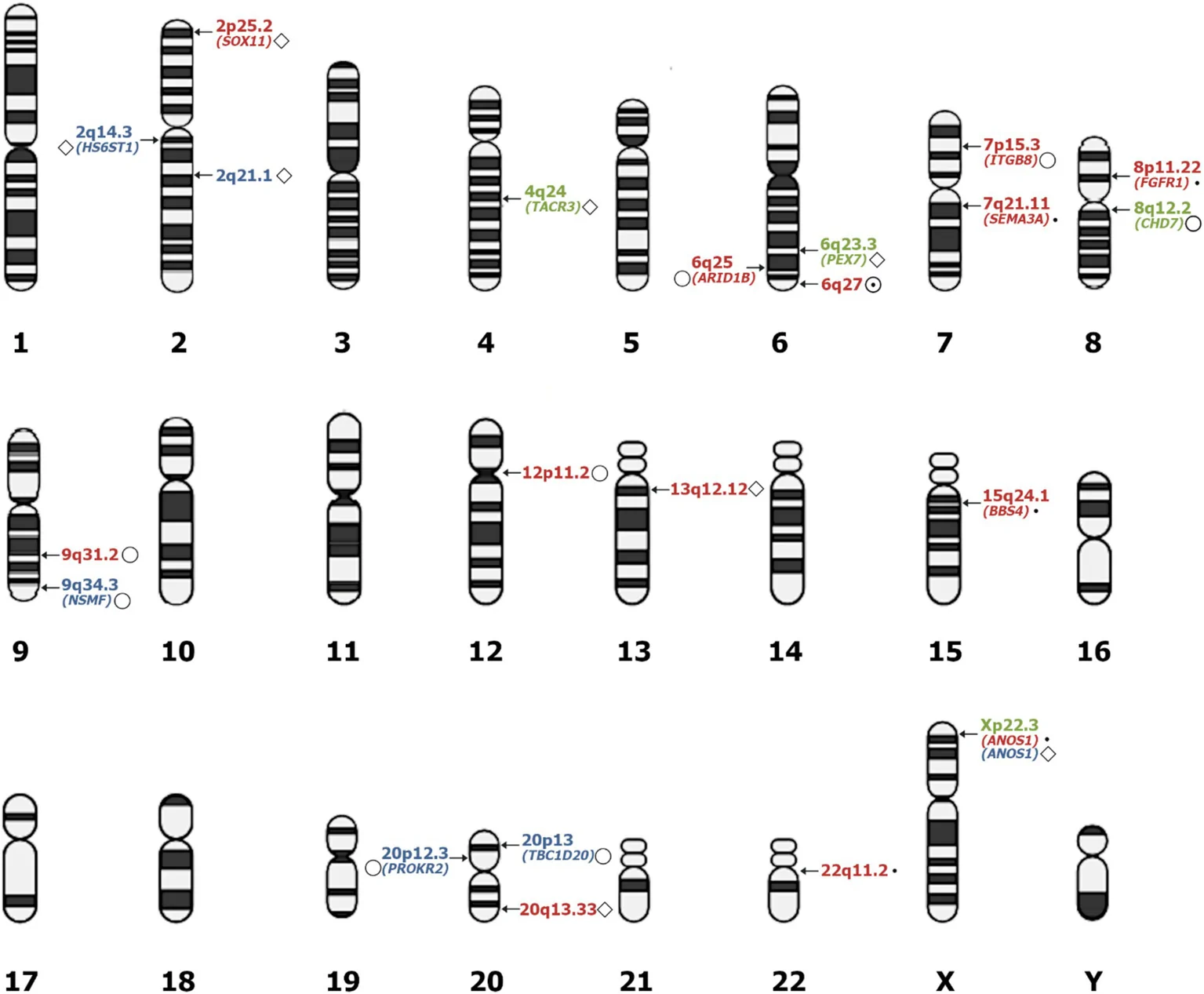
Chromosomal distribution and evidence strength of CNV *loci* evaluated in relation to congenital or syndromic olfactory dysfunction. Chromosomal *loci* are shown according to CNV type: red indicates deletions, blue indicates duplications, and green indicates regions in which both deletions and duplications have been reported. Symbols indicate the strength of evidence for the CNV-olfactory dysfunction association according to the framework defined in Table 1: filled circle, established; partially filled circle, probable; open circle, possible; and diamond, speculative. Candidate genes shown next to each *locus* represent the most biologically plausible gene(s) within the CNV interval and should not necessarily be interpreted as the sole cause of the olfactory phenotype, particularly for large multigenic rearrangements. Where deletions and duplications at the same locus were assigned different evidence levels, separate symbols are shown for each CNV type.

**Table 1:** Evidence-level framework for evaluating CNV-olfactory dysfunction associations.

Evidence level	Definition
Established	Human CNV associated with confirmed olfactory dysfunction and supported by a strong and/or replicated genotype-phenotype relationship, together with independent human genetic, mechanistic, or functional evidence implicating the relevant gene or *locus* in olfactory development or function.
Probable	Human CNV associated with anosmia/hyposmia or olfactory bulb/tract aplasia or hypoplasia, but supported by a limited number of reported cases and/or involving a multigenic CNV that complicates attribution to a single candidate gene.
Possible	Association based on a single or small number of cases, self-reported olfactory dysfunction, neuroimaging findings, or limited clinical phenotyping; the implicated gene or *locus* is biologically plausible, but independent replication is lacking.
Speculative	No documented olfactory dysfunction in the CNV carrier, or the association is based primarily on database annotation, gene expression, animal models, functional data, or biological plausibility without direct human olfactory evidence.

## 2 How copy number variants might shape olfactory phenotypes

Microdeletions and microduplications, collectively referred to as CNVs, are submicroscopic chromosomal abnormalities involving the loss or gain of DNA segments, typically ranging from a few kilobases to several megabases (Shaikh, 2017). While CNVs are often undetectable by conventional cytogenetic methods, they can be readily identified using high-resolution genomic approaches, such as array comparative genomic hybridization (aCGH) and next-generation sequencing (NGS; Pinkel et al., 1998; Weise et al., 2012). These technologies have revealed recurrent CNVs associated with or without well-defined syndromes, yet their contribution to olfactory disorders remains underrecognized. The molecular mechanisms by which CNVs exert their effects are diverse and often *locus*-dependent (Nowakowska, 2017). In the context of olfactory dysfunction, CNVs may contribute to the phenotype through several mechanisms:

- Gene dosage—altered copy number of dosage-sensitive genes can disrupt the delicate balance of molecular pathways required for olfactory system development. For example, deletions may cause haploinsufficiency, whereas duplications may result in overexpression with equally detrimental effects.
- Gene interruption—CNVs that partially delete or duplicate a gene may truncate coding sequences or disrupt splicing, resulting in nonfunctional proteins or aberrant transcripts.
- Gene fusion—CNVs may juxtapose exons from two different genes, creating fusion transcripts with unpredictable functional consequences.
- Position effects—structural changes can relocate genes relative to regulatory elements, altering their expression patterns without directly affecting the coding sequences (Freeman et al., 2006; Feuk et al., 2006).
- Unmasking of recessive alleles—deletion of one allele may expose pathogenic variants in the remaining allele, particularly in autosomal recessive genes associated with syndromic anosmia (Yuan et al., 2020; Louden et al., 2021).
- Regulatory disruption and transvection effects—Noncoding CNVs can affect long-range regulatory elements or allele-allele communication, leading to altered expression in genes essential for olfactory bulb development or odorant receptor signaling (Zhang et al., 2009; Guo et al., 2024).

The interpretation of CNVs in olfactory disorders raises several challenges. First, distinguishing pathogenic from benign CNVs is difficult due to incomplete penetrance, variable expression, and the frequent occurrence of CNVs in the general population (Nowakowska, 2017). Second, phenotypic variability among individuals carrying the same CNV complicates genotype-phenotype correlations, suggesting the presence of genetic modifiers, epigenetic influences, or environmental interactions (Freeman et al., 2006; Girirajan and Eichler, 2010). Finally, many candidate genes within olfactory-related CNVs remain poorly characterized in humans, and much of the knowledge derives from animal models, which may not fully reflect human biology (Freeman et al., 2006).

Taken together, these mechanisms underscore the complexity of interpreting CNVs in olfactory disorders. While gene dosage and direct gene disruption provide the most intuitive explanations, regulatory and positional effects may be equally relevant in explaining subtle or variable phenotypes. Systematic analysis of CNVs associated with olfactory dysfunction, integrating genomic content with functional annotation, is essential for expanding our understanding of the molecular architecture of human olfaction. This knowledge may ultimately inform diagnostics and guide the development of targeted therapies for individuals affected by congenital or syndromic loss of smell.

## 3 Literature and database search strategy and evidence assessment

We conducted a structured literature and database review to identify copy number variants potentially associated with congenital or syndromic olfactory dysfunction. PubMed, OMIM, DECIPHER, ClinVar, LOVD, and GEO were queried using database-specific search strategies, and access dates are provided in Supplementary Table S1. Searches combined terms related to structural variation (“copy number variant,” “CNV,” “microdeletion,” “microduplication,” “deletion,” “duplication”) with olfactory phenotypes (“anosmia,” “hyposmia,” “olfactory dysfunction,” “olfactory bulb aplasia,” and “olfactory bulb hypoplasia”) or candidate CNV regions/genes identified through literature and phenotype searches.

Records were considered eligible for the primary CNV-olfactory dysfunction evidence set when they described a human CNV and either: (i) documented anosmia or hyposmia using formal olfactory testing; (ii) reported anosmia or hyposmia based on clinical history or self-report; or (iii) demonstrated olfactory bulb or tract aplasia/hypoplasia by neuroimaging. CNVs involving genes or *loci* with established or experimentally supported biological relevance to olfactory development, but without documented olfactory assessment in the CNV carrier, were retained separately as hypothesis-generating observations. These records were not considered direct evidence of an association between CNV and the olfactory phenotype.

We excluded acquired olfactory disorders, sequence variants without a CNV component, and CNVs without sufficient genomic or phenotypic information for interpretation. Sequence variants were considered only as supporting biological or disease-related evidence for genes located within CNV regions and were not included as primary CNV observations.

Olfactory evidence was categorized as validated when assessed using a standardized psychophysical test; imaging-supported, when olfactory bulb or tract abnormalities were demonstrated by neuroimaging without validated smell testing; clinically reported, when anosmia or hyposmia was documented based on medical history or self-report; and not assessed, when no information on olfactory function was available.

To distinguish well-supported associations from candidate observations, *loci* were additionally classified into four evidence categories: established, probable, possible, and speculative (Table 1). Classification considered the presence and method of olfactory phenotyping, the number of independent affected individuals, recurrence of the CNV-phenotype association, CNV size and gene content, human genetic evidence implicating the candidate gene in olfactory biology, and supporting functional or experimental evidence. Importantly, this evidence classification refers specifically to the strength of the association between the CNV and olfactory dysfunction and is independent of the clinical pathogenicity classification assigned to the CNV by ClinVar, DECIPHER, or the original publication.

## 4 Established CNV-olfactory dysfunction associations

The *loci* included in this category are supported by direct human evidence of olfactory dysfunction in CNV carriers, together with replicated genotype-phenotype observations and/or independent functional or mechanistic data. Although the strength and nature of the supporting evidence differ between *loci*, these associations were considered sufficiently robust to be classified as established, while acknowledging variable penetrance and phenotypic expressivity.

### 4.1 7q21.11 deletion

In an extensive study involving a cohort of 120 patients of both sexes diagnosed with KS, the *SEMA3A* gene was identified as contributing to the molecular pathogenesis of the disorder (Young et al., 2012, Supplementary Table S2). From this cohort, 48 individuals without pathogenic variants in known KS genes were selected for further analysis. Comparative genomic hybridization using Agilent 105K oligonucleotide arrays identified a heterozygous 213 kb deletion at *locus* 7q21.11 in a male patient with KS and complete anosmia. The deletion, confirmed by quantitative real-time PCR, encompassed 11 of the 17 exons of *SEMA3A*. No additional neurological or systemic abnormalities were reported in this patient. A similar finding was reported in a male patient with KS and anosmia carrying a heterozygous ~236 kb deletion encompassing the entire *SEMA3A locus* (Stamou et al., 2022, Supplementary Table S2). The clinical phenotype additionally included learning difficulties, pes cavus, and flat feet, further broadening the phenotypic spectrum associated with *SEMA3A* haploinsufficiency. Additional CNV evidence was provided by Gileta et al. (2021), who described a female patient with skeletal dysplasia, hypogonadotropic hypogonadism, and anosmia carrying biallelic loss-of-function variants in *SEMA3A*. The patient harbored the nonsense variant c.1633C > T (p.Arg555*) in trans with a deletion encompassing exons 15–17 of *SEMA3A*. CNV analysis of targeted NGS data identified the three-exon deletion, which was subsequently confirmed using a custom Multiplex Ligation-Dependent Probe Amplification (MLPA) assay. Both variants were interpreted as pathogenic. Importantly, the patient's anosmia was supported by neuroimaging demonstrating the absence of the olfactory nerves. This observation provides additional human evidence linking structural loss of *SEMA3A* to combined olfactory and reproductive dysfunction, although in this case the phenotype resulted from biallelic *SEMA3A* loss of function rather than isolated heterozygous haploinsufficiency.

SEMA3A is a well-established member of the secreted semaphorin family that signals primarily through neuropilin-containing receptor complexes (de Castro, 2009). SEMA3A plays an important role in cell migration and axonal guidance during central nervous system (CNS) development and was among the first semaphorins implicated in the formation of the lateral olfactory tract (de Castro et al., 1999; Schwarting et al., 2000; Giacobini, 2015). Additional supporting evidence comes from Nrp1(*sema/sema*) mutant mouse models, which lack a functional semaphorin-binding domain in neuropilin-1 and show abnormal olfactory system development and defective embryonic migration of gonadotropin-releasing hormone (GnRH) neurons, providing a mechanistic parallel to the human KS phenotype (López-Mascaraque and de Castro, 2002; Cariboni et al., 2011; Hanchate et al., 2012).

Nevertheless, the clinical consequences of *SEMA3A* loss are heterogeneous, and olfactory dysfunction is not uniformly observed among individuals with pathogenic deletions, indicating incomplete penetrance and variable expressivity of the olfactory phenotype.

### 4.2 8p11.22 deletion

Deletions involving the 8p11.22 region may encompass multiple genes, among which *FGFR1* represents the principal candidate for the olfactory and neuroendocrine phenotype. *FGFR1* encodes a fibroblast growth factor receptor essential for embryonic development, including skeletal formation and the establishment of the olfactory and neuroendocrine systems. It plays a key role in the development of the olfactory placode and in shaping the migratory environment guiding GnRH neurons toward the hypothalamus (Murcia-Belmonte et al., 2010). Disruption of *FGFR1* signaling impairs olfactory bulb development and indirectly affects GnRH neuronal migration, leading to congenital anosmia or hyposmia in the context of KS. Notably, haploinsufficiency of *FGFR1* has been associated with abnormalities in olfactory system formation and reproductive hormone regulation (Chung et al., 2008). Evidence from functional assays and animal models further indicates that, while FGFR1 signaling within GnRH neurons themselves is not strictly required for pubertal onset or maintenance, it is critical for earlier developmental processes that coordinate the formation of the olfactory system and the proper migration of GnRH neurons (Dela Cruz et al., 2021).

Additional clinical reports highlight the broad phenotypic spectrum associated with deletions involving the 8p11 region. Although *FGFR1* is considered the major candidate gene implicated in KS and olfactory dysfunction, the frequently multigenic nature of these rearrangements should be taken into account when interpreting other clinical manifestations. For example, three patients with KS showed complex and variable clinical manifestations: patient #7 had spherocytosis, bent digits, color blindness, other eye disorders, cleft lip/palate, crowded teeth, double uvula, missing teeth, peripheral neuropathy, and speech impairment; patient #8 had KS with alopecia; finally, patient #9 had KS with shortened fourth metacarpals (Stamou et al., 2022, Supplementary Table S2). Furthermore, deletions involving *FGFR1* have been associated with anosmia, but their sizes exceeded those of typical microdeletions and therefore were not included in Supplementary Table S2. Notable examples include a chr8p11.1-p12 deletion associated with anosmia, bilateral cryptorchidism, micropenis, spherocytosis, growth delay (GD), and mild developmental delay (Cau et al., 2005), and a chr8p11.2 deletion encompassing *FGFR1* and *ANK1* in a KS patient with spherocytosis, GD, micrognathia, and bilateral crumpled ears (Vermeulen et al., 2002). Additionally, patient #6 reported by Stamou et al. (2022) presented with KS and anosmia in the context of a deletion larger than 9.6 Mb, accompanied by strabismus, hypodontia, and hearing loss.

### 4.3 15q24.1 deletion

A homozygous ~6 kb deletion at 15q24.1, removing exons 3 and 4 of the *BBS4* gene, has been reported in members of a five-generation Italian family affected with Bardet-Biedl syndrome (BBS, Iannaccone et al., 2005). Besides the core clinical manifestations of BBS (retinal degeneration, syndactyly, obesity, dental anomalies, cryptorchidism, and renal changes), the affected individuals showed clear evidence of olfactory dysfunction. Formal testing with the University of Pennsylvania Smell Identification Test (UPSIT) demonstrated anosmia in one patient and severe hyposmia/microsmia in a distant cousin, both values well below the 2nd centile (Supplementary Table S2).

These findings provide direct clinical evidence that *BBS4* plays a role in regulating olfactory function. They agree with previous observations that several BBS proteins, including BBS4, are expressed in the olfactory epithelium and that mouse models affecting several *BBS*-related genes exhibit profound olfactory deficits (Kulaga et al., 2004; Fath et al., 2005). These findings support ciliary dysfunction as an important mechanism underlying olfactory impairment in BBS and demonstrate that smell dysfunction may represent an under-recognized component of the phenotype.

### 4.4 22q11.2 deletion

A recurrent heterozygous deletion at chromosome 22q11.2, typically spanning ~1.5-–2.54 Mb, encompasses multiple dosage-sensitive genes implicated in CNS development and olfactory processing, including *COMT, PRODH, DGCR8*, and *CRKL* (Supplementary Table S2). Notably, *COMT* and *PRODH* are involved in neurotransmitter metabolism (dopamine, glutamate, and GABA) and contribute to cortical-subcortical circuits relevant to olfaction (McDonald-McGinn et al., 2015), providing a plausible genetic basis for the high prevalence of olfactory dysfunction.

This genomic region underlies the classical 22q11.2 deletion syndrome (22q11DS), also known as DiGeorge syndrome or velocardiofacial syndrome, which occurs in approximately 1 in 4,000-–5,000 live births and is associated with congenital malformations, immunodeficiency, and an increased risk of cognitive and psychiatric disorders (McDonald-McGinn et al., 2015).

Assessment using the UPSIT has shown that up to 68% of children with 22q11DS exhibit olfactory deficits, compared with 13% of their unaffected siblings. These impairments are not attributable to general intellectual disability, although they may be influenced by velopharyngeal insufficiency and altered nasal airflow (Sobin et al., 2006). Consistent with these findings, subsequent studies have demonstrated that deficits in odor identification and discrimination are features of 22q11DS and largely independent of global cognitive impairment (Tang et al., 2018).

### 4.5 Xp22.3 deletion

Advances in molecular diagnostic techniques have revealed numerous deletions in the Xp22.3 region, encompassing the *ANOS1* gene (formerly known as *KAL1*), strongly associated with anosmia and HH, the hallmarks of KS (Supplementary Table S2; de Castro et al., 2017). Not only *ANOS1* but also neighboring genes (*STS, PNPLA4, NLGN4X*, and members of the *VCX* family) have been repeatedly reported in patients with KS. Among the deleted genes, *ANOS1* is a critical determinant of the olfactory phenotype, as it encodes anosmin-1, a protein essential for the proper development and interconnection of olfactory structures and migration of GnRH neurons during embryonic development (Schwanzel-Fukuda and Pfaff, 1989; Dodé and Hardelin, 2009; Hardelin et al., 1999; Soussi-Yanicostas et al., 2002; López-Mascaraque and de Castro, 2002). Importantly, anosmin-1 exerts its function through protein-protein interactions within the extracellular matrix, notably modulating FGF signaling via interaction with FGFR1 (Murcia-Belmonte et al., 2010; Esteban et al., 2013). In the olfactory system, this interaction is crucial for the guidance and targeting of olfactory axons as well as for the coordinated migration of GnRH neurons along olfactory pathways. Disruption of the anosmin-1-FGFR1 functional axis may therefore contribute to the variability and graded severity of olfactory and reproductive phenotypes observed in patients with KS.

Classic studies documented intragenic deletions of *ANOS1* in families with X-linked KS (Franco et al., 1991; Legouis et al., 1991; Bick et al., 1992), with affected males typically showing anosmia or severe hyposmia, delayed puberty, and hypogonadism, while carrier mothers were unaffected. In one family, a 3.3 kb deletion confined within the 210 kb *ANOS1* revealed a short 6 bp homology (CAAATT), suggesting that nonhomologous recombination may underlie such intragenic events, as previously postulated (Woods-Samuels et al., 1991). Notably, copies of this 6 bp motif are present in the intronic sequences flanking the exon 5–10 deletion reported by Trarbach et al. (2005) and the intragenic deletion described by Nagata et al. (2000). Thus, this mechanism may contribute to the recurrence of this specific intragenic deletion.

Larger contiguous deletions extending from *ANOS1* into *STS* have also been reported, resulting in combined phenotypes such as KS with X-linked ichthyosis, highlighting the clinical variability associated with structural rearrangements in Xp22.3 (Maya-Núñez et al., 1998; Xu et al., 2015). More recent studies (e.g., Kim et al., 2023) have expanded the spectrum of *ANOS1* defects, demonstrating that both point mutations and CNVs, including exon-level and whole-gene deletions, contribute substantially to KS and normosmic IHH (Kim et al., 2023). *ANOS1* defects accounted for approximately 14% of cases in their cohort, underscoring the clinical relevance of this *locus*. Additional features such as renal agenesis, cryptorchidism, or synkinesis may co-occur, but anosmia remains the most consistent and defining manifestation. These observations confirm that deletions in Xp22.3 encompassing *ANOS1* represent a well-established molecular basis for congenital anosmia. Therefore, routine screening of this *locus*, including CNV analysis, is recommended in patients with suspected KS or ICA.

## 5 Probable CNV-olfactory dysfunction associations

Clinically relevant human observations support the associations grouped in this category, but the available evidence remains insufficient for definitive classification. Their interpretation is therefore based on converging yet still limited clinical and biological data and should be refined as additional well-phenotyped cases become available.

### 5.1 6q27 deletion

These are large terminal deletions of the distal long arm of chromosome 6 (~5.21 Mb and ~5.30 Mb). The deleted interval encompasses multiple genes implicated in neurodevelopment, including *DLL1, PHF10, C6orf70 (ERMARD)*, and *TBP*. Haploinsufficiency of one or more of these genes is thought to underlie the structural brain anomalies, developmental delay, and other features observed in 6q27 microdeletion syndrome. In two unrelated individuals (one adult, one child), high-resolution MRI revealed hydrocephalus, midline brain anomalies, and bilateral aplasia of the olfactory bulbs (Gerber et al., 2011, Supplementary Table S2): while the adult patient (25 years old) had clinically confirmed anosmia, anosmia was strongly suspected based on neuroimaging findings in the 3-year-old child. Olfactory bulb aplasia and anosmia had not been widely recognized features of 6q27 microdeletion before this report, and the authors proposed these as part of the expanded phenotype. Given that anosmia can affect safety and quality of life, these authors recommend targeted smell testing and MRI assessment in patients with terminal 6q27 deletions (Gerber et al., 2011). Over two dozen patients with terminal 6q27 deletions of similar size have been described in the literature and the DECIPHER database. While there is a consistent association with brain malformations, ID, and, in some cases, seizures, the prevalence of anosmia is uncertain and may be underreported. Clinical correlation, olfactory testing, and family studies are recommended for any new cases to refine the understanding of genotype-phenotype relationships.

## 6 Possible CNV-olfactory dysfunction associations

The *loci* included in this category are supported by preliminary human observations suggesting a potential relationship with olfactory dysfunction, but the available evidence remains limited and heterogeneous. These associations should therefore be interpreted cautiously and regarded as provisional until supported by independent replication, more detailed olfactory phenotyping, or additional functional evidence.

### 6.1 6q25 deletion

Haploinsufficiency of *ARID1B*, a member of the SWI/SNF chromatin-remodeling complex, is a frequent cause of ID and is associated with Coffin-Siris syndrome (CSS; Hoyer et al., 2012). While the primary clinical features include ID, speech impairment, and facial dysmorphisms, some patients exhibit absent olfactory bulbs and anosmia (Nevado et al., 2014). A 6q25.3 deletion encompassing *ARID1B* was also reported by Lu et al. (2021), although olfactory function was not assessed in that patient. Additionally, a patient with a microduplication/microdeletion involving *ARID1B*, as described by Hoyer et al. (2012), exhibited ID and growth delay; however, anosmia was not reported.

*ARID1B* plays an important role in chromatin remodeling and neuronal connectivity during neurodevelopment (Ka et al., 2016). However, formal psychophysical assessment of smell was not reported, and because *ARID1B*-associated CNVs may occur within broader neurodevelopmental rearrangements, a direct dosage-dependent effect on olfactory function remains difficult to distinguish from more general developmental abnormalities. Thus, current human evidence linking *ARID1B* CNVs to olfactory dysfunction remains limited.

### 6.2 7p15.3 deletion

This is a microdeletion of approximately 93.5 kb on chromosome 7p15.3, encompassing the *ITGB8* gene, which encodes integrin β8, a receptor subunit involved in cell-extracellular matrix interactions, TGF-β activation, and key developmental processes in the CNS, craniofacial structures, and inner ear. Very recently, it has been suggested that this gene plays a role in microglial development (McKinsey et al., 2025). The DECIPHER patient (#389940, Supplementary Table S2) exhibits ear anomalies with severe hearing impairment, HH, ophthalmoplegia, choanal atresia, and anosmia. These findings are consistent with *ITGB8*’s roles in neural (including auditory and visual systems) and craniofacial development, with anosmia potentially reflecting a combination of nasal malformation and olfactory pathway abnormalities. Although clinically relevant *ITGB8* variants have been associated with developmental anomalies, small deletions of this size are rare, and pathogenicity remains uncertain.

### 6.3 8q12.2 deletion/duplication

Multiple structural variants involving the *CHD7* gene at chromosome 8q12.2 have been reported. A heterozygous 5.3 kb deletion affecting exon 35 of *CHD7* has been classified as likely pathogenic, predicted to disrupt part of the Brahma and Kismet (BRK) domain, and is consistent with loss-of-function as a known disease mechanism. This variant has been associated with HH5 with or without anosmia. Larger rearrangements further support the involvement of *CHD7* in olfactory dysfunction: a 2.3 Mb *de novo* deletion at 8q12.2 was identified in two individuals with CHARGE syndrome (Vissers et al., 2004), while additional DECIPHER entries document 4.77 Mb (of uncertain significance) and a 4.39 Mb pathogenic deletion (Supplementary Table S2). Although *CHD7* CNVs are generally considered to contribute to only a minority (<5%) of CHARGE syndrome cases, published evidence suggests that they do occur and may be clinically relevant. For example, a 4-year-old girl was reported with a *de novo* 6.9 Mb tandem duplication including *CHD7*, presenting with hypotonia, ID, Duane anomaly, and congenital heart defects (Lehman et al., 2009, Supplementary Table S3). Olfactory function was not reported. A similar case with overlapping microduplication at 8q12 involving *CHD7* was previously described by Monfort et al. (2008). These reports suggest that abnormal *CHD7* dosage, including both deletions and duplications, may underlie a recurrent constellation of developmental and sensory anomalies. However, the contribution of duplications to isolated HH (IHH) or anosmia remains uncertain.

*CHD7* encodes the chromodomain helicase DNA-binding protein 7, a chromatin remodeler that plays critical roles in neural crest development, sensory organ formation, and olfactory system development. Experimental evidence indicates that *CHD7* regulates neural progenitor proliferation in the olfactory placode and influences the expression of key developmental genes, including *FGFR1, BMP4*, and *OTX2*, thereby contributing to proper neurogenesis and the establishment of GnRH neurons (Layman et al., 2011). Pathogenic variants in *CHD7* are the primary cause of CHARGE syndrome, in which anosmia or hyposmia is frequently observed. These olfactory deficits are thought to arise not only from abnormal olfactory bulb development but also from impaired neurogenesis and reduced numbers of GnRH neurons, likely secondary to disrupted progenitor proliferation and altered developmental signaling within the olfactory epithelium (Layman et al., 2011).

### 6.4 9q31.2 deletion

A patient has been reported with congenital hypogonadotropic hypogonadism (CHH) and anosmia carrying a *de novo* 2.38 Mb heterozygous deletion in 9q31.2 (Iivonen et al., 2021, Supplementary Table S2). The deletion encompassed six protein-coding genes: *FKTN, ZNF462, TAL2, TMEM38B, RAD23B*, and *KLF4*. Haploinsufficiency of *ZNF462* was consistent with the patient’s Weiss-Kruszka syndrome phenotype (craniofacial anomalies, ID, and sensorineural hearing loss), but did not explain his KS. Whole-exome and RNA sequencing excluded variants or splicing defects in known KS genes, and no suppression of the nearby candidate *locus PALM2AKAP2* was observed. In addition, the DECIPHER database includes a patient (#371038) with a 487.97 kb deletion affecting *FKTN, TAL2*, and *TMEM38B*, which is associated with HH. These findings suggest that microdeletions in 9q31.2 may predispose to KS/CHH, and olfactory/reproductive phenotypes should be carefully explored in patients carrying such deletions.

### 6.5 9q34.3 duplication

A *de novo*, heterozygous microduplication of ~178 kb at 9q34.3 has been identified in a male patient with KS (Stamou et al., 2022, Supplementary Table S3). This multigenic duplication includes the *NSMF* gene (NMDA Receptor Synaptonuclear Signaling and Neuronal Migration Factor, also known as NELF). *NSMF* has been proposed as a guidance cue important for olfactory/vomeronasal axon patterning and GnRH neuron migration during embryonic development. Knockdown studies in mice and zebrafish demonstrated defective GnRH neuron migration and abnormal olfactory/vomeronasal nerve projections (Kramer and Wray, 2000; Palevitch et al., 2009). In contrast, *Nsmf-*null mutant mice show sexually dimorphic reproductive phenotypes (Quaynor et al., 2015). However, the role of *NSMF* in human disease remains less clear. Variants in *NSMF* (9q34.3) have been reported in both KS and normosmic CHH, often in an oligogenic context, raising questions about their pathogenicity. In this case, the patient exhibited synkinesis, scoliosis, and bone abnormalities in addition to anosmia. Although most reported variants involve potential loss-of-function mechanisms, this finding suggests that gene dosage alterations, such as duplications, may also contribute to disease pathogenesis. Further studies are required to clarify whether *NSMF* duplications contribute directly to the pathogenesis of KS or other olfactory disorders, or whether they act in combination with other genetic factors.

### 6.6 12p11.2 deletion

CGH array identified a heterozygous 4.7 Mb deletion at 12p11.21-12p11.23 in a patient with KS and ID (Ben-Mahmoud et al., 2023). The deletion was detected in the context of an apparently balanced *de novo* reciprocal translocation, described initially as t(7;12)(q22;q24)dn and later refined as t(7;12)(q21.13;q23.1)dn, but occurred independently of the translocation breakpoints. This early adult man presented with delayed sexual maturation, HH (low FSH, LH, testosterone), anosmia, small testes, and skeletal anomalies such as brachydactyly and delayed bone age. The deleted interval encompasses several candidate genes implicated in KS and/or neurodevelopmental disorders. Among them, *TSPAN11* has been proposed as a KS candidate, while *INTS13, REP15, PPFIBP1*, and *FAR2* may contribute to combined KS with ID. Other genes in this region (e.g., *TM7SF3, STK38L, ARNTL2, ERGIC2, TMTC1, DENND5B, ETFBKMT*) have been linked to neurodevelopmental phenotypes (Ben-Mahmoud et al., 2023). Expression studies have demonstrated that many of these genes are highly expressed in the brain and reproductive tissues, supporting their role in olfactory and reproductive functions. Together with additional CNVs involving the 12p11.21-p11.23 region reported in public databases such as DECIPHER, these findings support that this *locus* is prone to recurrent copy number changes. Although most reported cases lack detailed phenotypic annotation for KS or anosmia, the gene content of this region and its expression profile suggest that dosage alterations may contribute to neurodevelopmental and reproductive phenotypes. However, further studies, including detailed genotype-phenotype correlation and sequencing of candidate genes, are required to clarify their potential role in the pathogenesis of KS, anosmia, and cognitive impairment.

### 6.7 20p12.3 duplication

A complete gene duplication of *PROKR2*, with a copy number increase of up to five copies (CN = 5), was identified in a male patient with Kallmann syndrome (Stamou et al., 2022, Supplementary Table S3). The duplication spanned approximately 13 kb and was heterozygous. The patient also presented with a cleft lip and palate. This observation provides direct human evidence linking increased *PROKR2* copy number with a KS phenotype, although it currently represents an isolated CNV observation.

The biological plausibility of this association is supported by the well-established involvement of *PROKR2* in olfactory bulb development and GnRH neuronal migration. Pathogenic sequence variants in *PROKR2* have been repeatedly reported in patients with KS and HH, as well as in individuals with isolated congenital anosmia (Dodé et al., 2006; Sarfati et al., 2010; Moya-Plana et al., 2013). Nevertheless, because evidence specifically for *PROKR2* copy-number gain is currently limited to a small number of observations, the contribution of increased *PROKR2* dosage to olfactory and reproductive phenotypes remains to be established.

### 6.8 20p13 duplication

A ~92 kb duplication on chromosome 20p13, spanning the *TBC1D20* gene, has been reported in a female patient initially ascertained through an IHH cohort (Stamou et al., 2022) but later reclassified as KS due to anosmia. In the cited report, the duplication was inherited from the patient’s mother, who had anosmia but a normal reproductive history (Stamou et al., 2022; Supplementary Table S3). This segregation suggests that altered *TBC1D20* dosage may contribute to olfactory dysfunction, whereas reproductive involvement may be variably penetrant or dependent on additional modifiers. The proband also displayed additional neurological features, including nystagmus, ophthalmoplegia, ptosis, speech impairment, and cerebral ataxia.

*TBC1D20* encodes a Rab GTPase-activating protein for RAB1 and RAB2 and plays a key role in endoplasmic reticulum function, Golgi morphology, and lipid droplet homeostasis. Pathogenic variants in this gene have been associated with Warburg Micro syndrome and related syndromic forms, including HH (Liegel et al., 2013). Notably, loss-of-function alterations in *TBC1D20* include not only point mutations but also structural variants. A homozygous microdeletion, encompassing exons 2–8, has been described in an affected individual and is predicted to result in complete loss of protein function, supporting a loss-of-function disease mechanism (Liegel et al., 2013). This observation further underscores the importance of proper *TBC1D20* dosage for normal cellular and developmental processes.

## 7 Speculative CNV-olfactory dysfunction associations

The *loci* included in this category are retained primarily as hypothesis-generating observations rather than as established CNV-olfactory associations. Their relevance is based on biological plausibility, database reports, or indirect experimental evidence, while direct human evidence of olfactory dysfunction is absent or insufficient.

### 7.1 2p25.2 deletion

Deletions involving *SOX11* at 2p25.2 have been reported in individuals with neurodevelopmental disorders. Hempel et al. (2016) described seven patients with *SOX11* deletions encompassing *SOX11* and features of Coffin-Siris syndrome, two of whom had intragenic microdeletions. Similarly, Al-Jawahiri et al. (2022) reported two additional microdeletions affecting this gene (Supplementary Table S2). Although anosmia was not documented, *SOX11* encodes a transcription factor essential for embryonic neurogenesis and neuronal differentiation (Hempel et al., 2016; Al-Jawahiri et al., 2022), suggesting its potential relevance to olfactory function. *SOX11* is expressed in telencephalic regions involved in olfactory bulb development (Wang et al., 2013), and *Sox11*-null mice exhibit hypoplastic olfactory bulbs (Sun et al., 2025). Furthermore, patients with *SOX11* variants may present with HH, with or without anosmia, consistent with disrupted migration of GnRH neurons originating in the olfactory placode (Chu et al., 2026). Both findings suggest that *SOX11* haploinsufficiency contributes to neurodevelopmental phenotypes and may impair olfactory pathway development.

### 7.2 2q14.3 duplication

The identified microduplication spans approximately 12.9 kb and includes the *HS6ST1* gene, which encodes a heparan sulfate 6-O-sulfotransferase (Supplementary Table S3). This enzyme plays a crucial role in neuronal development, including the formation of the olfactory system and axon branching (Tornberg et al., 2011). Autosomal dominant pathogenic variants in *HS6ST1* have been associated with HH15, with or without anosmia (Tornberg et al., 2011; OMIM #614880). Although the duplication observed here is relatively small, *HS6ST1* represents a candidate gene that may be involved in olfactory dysfunction. To date, we have identified at least 26 cases of duplication in this region across medical databases and the scientific literature, though they typically involve larger segments. The clinical relevance of this duplication remains to be clearly determined, both in its resulting symptomatology and in its genetic prevalence. However, given the gene’s function and disease associations, this region may represent a *locus* of interest for olfactory disorders.

### 7.3 2q21.1 duplication

Duplications of approximately 469 kb in the 2q21.1 region (chr2: 130724411-131193847, GRCh38) have been reported in three patients in the DECIPHER database. These duplications encompass six protein-coding genes: *AMER3, ARHGEF4, FAM168B, GPR148, PLEKHB2*, and *SMIM39*. One patient (DECIPHER #255882, male, 14 years) presented with anosmia, focal impaired awareness seizures, arachnodactyly, and scoliosis. Another (DECIPHER #290112) exhibited brachycephaly and microcephaly, while a third (DECIPHER #538902) had a similarly sized duplication, but no clinical data were available. Although the pathogenic significance of these duplications remains uncertain, the presence of anosmia and seizures in one patient and microcephaly in another suggests that this region may be involved in CNS development and olfactory processing. Further studies are needed to confirm the potential roles of other genes (*ARHGEF4, GPR148*) encoded in this chromosomal region in CNS development, particularly in the olfactory system.

### 7.4 4q24 deletion/duplication

The CNVs at 4q24 include the *TACR3* gene, which encodes the neurokinin 3 receptor (NK3R), a G-protein-coupled receptor involved in the development and function of GnRH neurons (Zhang et al., 2020). Pathogenic variants in *TACR3* are well-established causes of CHH, predominantly with preserved olfactory function (Topaloglu et al., 2009; Young et al., 2010; de Castro, 2009). While direct evidence in humans linking CNVs to olfactory dysfunction is limited, animal studies demonstrate that *Tacr3* is expressed in the olfactory bulb and other sensory pathways (Navarro et al., 2013; Zhang et al., 2020), supporting a potential role in olfactory system development. In DECIPHER, 17 CNVs overlapping this region have been reported, including 29% microduplications and 71% microdeletions. The majority span 1–10 Mb, while 12% are smaller (0.1–1 Mb), and phenotypes in multiple patients include intellectual disability and moderate postnatal growth retardation (Supplementary Tables S2, S3). Although larger CNVs often involve additional genes that complicate interpretation, *TACR3* is a plausible candidate contributing to neurodevelopmental phenotypes and potentially to olfactory dysfunction.

### 7.5 6q23.3 deletion/duplication

Copy-number changes involving the 6q23.3 region range from small intragenic rearrangements to large multigenic deletions and duplications. In ClinVar, 4 duplications have been reported, ranging in size to ~93 kb, with most involving the *PEX7* gene. In the DECIPHER database, eight duplications spanning this *locus* are larger (3.57–170 Mb), including *PEX7* and other genes, making the clinical picture uncertain. Deletions are more frequent, with 11 ClinVar entries and at least 22 DECIPHER patients. Clinical features include global developmental delay, autism spectrum disorder, hyperactivity, sleep disturbances, impaired pain sensation, and self-injurious behavior. One example (DECIPHER #524555, Supplementary Table S2) involves a 3.85 Mb deletion that affects 25 genes, including *PEX7*, which is associated with neurodevelopmental delay, sensory dysfunction, and sleep abnormalities. *PEX7* encodes the cytosolic receptor for peroxisomal matrix enzymes targeted by the PTS2 signal (Pan et al., 2013). Pathogenic variants in *PEX7* cause peroxisome biogenesis disorders, including adult Refsum disease (ARD), which is characterized by elevated plasma phytanic acid levels, late childhood- or adult-onset retinitis pigmentosa, and variable combinations of anosmia, polyneuropathy, deafness, ataxia, and ichthyosis. Some of these manifestations may be explained by the consequences of impaired peroxisomal function in the oligodendroglial lineage (van den Brink et al., 2003; Baes and Aubourg, 2009). Symptom onset can range from infancy to older than 50 years, with possible later-life complications such as cardiac arrhythmia and cardiomyopathy. Given the association of *PEX7* loss with anosmia in ARD, alterations in this region seem relevant to olfactory dysfunction and broader neurological and systemic manifestations.

### 7.6 13q12.12 deletion

A heterozygous 1.35 Mb deletion at chromosome 13q12.12 has been reported in a DECIPHER patient (#501263) presenting anosmia, with no additional phenotypic details provided. The deletion encompasses seven protein-coding genes: *C1QTNF9, C1QTNF9B, MIPEP, SACS, SGCG, SPATA13*, and *TNFRSF19*. None of these genes has a well-established role in the development or function of the olfactory system. *MIPEP* encodes a mitochondrial intermediate peptidase involved in the maturation of oxidative phosphorylation proteins and is highly expressed in energy-demanding tissues such as the heart, skeletal muscle, and pancreas (Wachoski-Dark et al., 2022). In contrast, *SGCG* encodes γ-sarcoglycan, a component of the dystrophin-associated glycoprotein complex that links the cytoskeleton to the extracellular matrix and is essential for muscle integrity and contractility (Crosbie et al., 2000). For *C1QTNF9, C1QTNF9B, SACS, SPATA13*, and *TNFRSF19*, no direct connection to smell perception has been established. The cause of anosmia in this patient, therefore, remains unclear. No gene within the deleted interval has an established role in human olfactory development or function. Therefore, the observed association remains hypothesis-generating, and no specific gene within the interval can currently be assigned to the olfactory phenotype. In summary, while the 13q12.12 deletion may contribute to anosmia in this patient, further studies are required to establish a causal link.

### 7.7 20q13.33 deletion

In addition, a ~531 kb deletion at 20q13.33 has been described in DECIPHER (patient #407019), encompassing multiple genes (*ARFGAP1, BIRC7, CHRNA4, COL20A1, EEF1A2, FNDC11, GMEB2, HELZ2, KCNQ2, NKAIN4, PPDPF, PTK6, RTEL1, SRMS, STMN3*, *TNFRSF6B*, and *YTHDF1*). The reported patient showed anosmia, delayed puberty, growth delay, speech/language impairment, and seizures. *ARFGAP1* is expressed in the human olfactory bulb according to the Human Protein Atlas and is involved in vesicle trafficking and neurotransmission, both of which are relevant to olfactory signal processing (Chornyy et al., 2017). Another gene, *CHRNA4*, encoding the α4 subunit of neuronal nicotinic acetylcholine receptors, is important for neural signaling (Dajas-Bailador and Wonnacott, 2004), although its direct role in olfaction is less well established.

### 7.8 Xp22.3 duplication

Duplications involving the Xp22.3 region, which encompasses the *ANOS1* gene, are reported far less frequently than pathogenic deletions, and their clinical significance remains unclear. Several small copy-number gains of *ANOS1* have been submitted to ClinVar, including events spanning the entire coding sequence or specific exons. Because their structural context is often unresolved and clinical data remain limited, most of these variants are currently classified as variants of uncertain significance (VUS; Oleari et al., 2021; ClinVar). Nevertheless, recent reports suggest that *ANOS*1 duplications may occasionally be associated with olfactory and reproductive phenotypes, although the available evidence remains insufficient to establish a definitive causal relationship.

Larger duplications in this region have been more systematically assessed in prenatal cohorts. Luo et al. (2026) analyzed fetuses carrying Xp22.31 copy number variants using a SNP array. Duplication sizes ranged from 1.29 Mb to 2.35 Mb, and the variants included multiple OMIM genes, such as *STS, ANOS1, DXS1283E, VCX3B*, and *HDHD1A*. Among the 11 fetuses with duplications, three (27.3%) presented with ultrasound anomalies, primarily cardiovascular malformations, ventriculomegaly, and polyhydramnios, whereas most showed no detectable abnormalities. Ten pregnancies were continued, and one was terminated; follow-up revealed only a single case of developmental delay affecting speech and motor milestones, while the remaining children appeared clinically unaffected.

These observations are consistent with other prenatal studies suggesting that most Xp22.31 duplications have limited clinical consequences (Hu et al., 2023). However, evidence from postnatal cohorts suggests that *ANOS1* duplications may not always be benign. Notably, Stamou et al. (2022) identified two patients with Kallmann syndrome and anosmia carrying duplications encompassing the *ANOS1 locus*, including one male and one female patient (Supplementary Table S3). Although these observations remain isolated and do not establish causality, they suggest that increased *ANOS1* dosage may contribute to olfactory and reproductive phenotypes in a subset of individuals.

It is also worth noting that a single case report suggested that *ANOS1* duplication and overexpression may lead to hyperosmia (Supplementary Table S3; Sowińska-Seidler et al., 2015). The proband presented with olfactory hypersensitivity, ectrodactyly, intellectual disability, genital anomalies, and dysmorphic features, together with markedly increased *ANOS1* transcript levels. However, this observation has not been replicated, and other studies of *ANOS1/*Xp22.3 duplications (Roumelioti et al., 2019) did not identify a consistent association with olfactory or syndromic phenotypes.

Overall, while loss-of-function variants and deletions of *ANOS1* remain the best-established genetic causes of anosmia and HH, the pathogenic significance of *ANOS1* duplications remains uncertain. Current evidence suggests that many Xp22.3 duplications have little or no apparent clinical consequence; however, the occurrence of *ANOS1*-containing duplications in patients with Kallmann syndrome indicates that dosage-sensitive effects cannot be completely excluded. Further functional studies and additional well-characterized clinical cases will be required to clarify the contribution of increased *ANOS1* dosage to olfactory, reproductive, and neurodevelopmental phenotypes.

## 8 Discussion

The evidence-stratified analysis revealed substantial differences in the strength of CNV–olfactory dysfunction associations across the identified *loci*. The strongest evidence was observed for deletions involving *SEMA3A*, the 8p11.22/*FGFR1* region, *BBS4*, 22q11.2, and Xp22.3/*ANOS1*, where human olfactory phenotypes are supported by recurrent observations and/or independent mechanistic evidence. In contrast, several other *loci* are supported only by isolated cases, database-derived observations, neuroimaging findings without functional smell testing, or indirect biological evidence. This distinction is important because biological plausibility or expression in olfactory structures alone should not be interpreted as evidence of a causal CNV-olfactory relationship.

Interpretation is particularly challenging for large multigenic CNVs. In such cases, olfactory dysfunction may reflect the combined effects of multiple dosage-sensitive genes or broader craniofacial and neurodevelopmental abnormalities, rather than the disruption of a single candidate gene. The 8p11.22 and 22q11.2 regions illustrate this complexity. In the former, *FGFR1* represents a strong candidate for the olfactory and neuroendocrine phenotype, whereas additional genes within larger deletions may contribute to other clinical manifestations. Similarly, olfactory dysfunction in 22q11.2 deletion syndrome may result from both central neurodevelopmental mechanisms and non-neural factors, including altered nasal airflow. Therefore, attributing the olfactory phenotype to a single gene within a multigenic CNV requires particular caution and, ideally, should be supported by focal CNVs, independent human genetic evidence, or functional studies.

Another major limitation of the available literature is the heterogeneity of olfactory phenotyping. Standardized psychophysical testing was available only for a subset of reported cases, whereas other observations relied on clinical history, self-report, or structural abnormalities detected by neuroimaging. Olfactory bulb or tract aplasia provides strong evidence of abnormal olfactory system development but should not be considered equivalent to psychophysically confirmed anosmia. Conversely, the absence of reported olfactory dysfunction cannot be interpreted as evidence of normal olfactory function when smell was not specifically assessed. This issue is particularly relevant for database-derived CNVs, in which phenotypic annotation is often incomplete. More systematic use of validated olfactory tests, together with dedicated assessment of olfactory bulb and tract morphology, would substantially improve genotype-phenotype correlations.

Despite the increasing availability of CNV databases and high-resolution genomic resources, the genetic architecture of congenital olfactory dysfunction therefore remains incompletely characterized. Rare microdeletions and microduplications can provide valuable insights into dosage-sensitive pathways involved in olfactory system development, but the strength of individual associations varies considerably. The evidence framework applied here was intended to separate well-supported CNV-olfactory relationships from provisional and hypothesis-generating observations, while retaining potentially informative *loci* that may warrant targeted phenotyping in future patients. Such an approach may be particularly useful in clinical genetics, where olfactory dysfunction is often underreported despite its potential value as a diagnostic clue.

The limited knowledge of the genetic basis of isolated congenital anosmia and broader olfactory phenotypes also represents an opportunity for further investigation. Integration of chromosomal microarray analysis with exome and whole-genome sequencing may identify additional structural and sequence-level variants and help distinguish primary dosage effects from oligogenic or modifying mechanisms. Importantly, future studies should combine genomic analysis with standardized olfactory phenotyping, neuroimaging, segregation analysis, and functional validation whenever possible. This integrated strategy may improve the interpretation of rare CNVs and clarify the molecular pathways underlying both isolated and syndromic olfactory dysfunction.

Overall, the available evidence supports an important but heterogeneous contribution of CNVs to congenital olfactory disorders. Rather than representing a uniform group of pathogenic alterations, these variants span a continuum from well-established dosage-sensitive *loci* to preliminary observations that remain primarily hypothesis-generating. Recognizing this distinction is essential for both clinical interpretation and future research and may facilitate more precise genotype-phenotype correlations in patients with congenital or syndromic olfactory dysfunction.
