# Supplementary material for: Olfactory dysfunction and submicroscopic abnormalities: unveiling hidden pathologies
Source: Front Mol Neurosci. 2026 Sep 30;19:1861373. doi: 10.3389/fnmol.2026.1861373 (PMC13662416; doi:10.3389/fnmol.2026.1861373)
Supplement: Supplementary file 1 [file Data_Sheet_1.PDF]

Supplementary Material

Supplementary Tables:

Supplementary Table S1. Database-specific search strategy and search terms used to identify CNVs potentially associated with olfactory dysfunction.

Database	Search/query strategy	Access date
PubMed	("copy number variant" OR CNV OR microdeletion OR microduplication OR deletion OR duplication) AND (anosmia OR hyposmia OR "olfactory dysfunction" OR "olfactory bulb aplasia" OR "olfactory bulb hypoplasia")	03 Sep 2026
OMIM	anosmia; hyposmia; olfactory dysfunction	18 Apr 2026
DECIPHER	anosmia; candidate CNV regions/genes identified through literature and phenotype search	18 Apr 2026
ClinVar	candidate CNV regions/genes identified through literature and phenotype search	18 Apr 2026
LOVD	candidate genes identified through literature and phenotype search	18 Apr 2026
GEO	anosmia; candidate CNV regions/genes identified through literature and phenotype search	18 Apr 2026

Supplementary Table S2. Characteristics of identified microdeletions, including chromosomal location, protein-coding gene content, variant size, clinical classification, method of olfactory dysfunction (OD) assessment or method not reported (NR), and olfactory phenotype (anosmia, hyposmia, or not assessed (NA)).

**Chr. region**	**Protein-coding genes**	**Length**	**Classification**	**Source/database**	**Diagnosis of OD**	**Olfactory phenotype**
2p25.2 2:5838893-7023548 (GRCh37)	*SOX11, RSAD2, CMPK2*	1,184,655 bp	Pathogenic	doi: 10.1136/jmedgenet-2015-103393 (patient no. 3)	NA	NA
2p25.2 2:5166592-6400126 (GRCh37)	*SOX11*	1,233,534 bp	Pathogenic	doi: 10.1136/jmedgenet-2015-103393 (patient no. 7)	NA	NA
2p25.1 2:5325900-10671580 (GRCh37)	*SOX11, ADAM17, ASAP2, CMPK2, CPSF3, CYS1, GRHL1, HPCAL1, IAH1, ID2, ITGB1BP1, KIDINS220, KLF11, MBOAT2, NOL10, ODC1, RNF144A, RRM2, RSAD2, TAF1B, YWHAQ*	5,345,680 bp	Uncertain significance	doi: 10.1016/j.gim.2022.02.013 (patient no. 20)	NA	NA

2p25.2 2:5831298-5836638 (GRCh37)	*SOX11*	5,340 bp	Uncertain significance	doi: 10.1016/j.gim.2022.02.013 (patient no. 24)	NA	NA
2p25.2 (2:5486129-5928416) (GRCh37)	*SOX11*	442,287 bp	Likely pathogenic	doi: 10.1038/s41431-024-01695-8 (patient no. 7)	NA	NA
4q24 4:99807055-104990025 (GRCh38)	*BANK1, BDH2, CENPE, CISD2, CXXC4, DAPP1, DDIT4L, DNAJB14, EMCN, H2AZ1, LAMTOR3,MANBA, NFKB1, PPP3CA, SLC39A8, SLC9B1, SLC9B2, TACR3, UBE2D3*	5,182,970 bp	Uncertain significance	DECIPHER: (patient no. 249234)	NA	NA
6q23.3 6: 136185822-140033807 (GRCh38)	*ABRACL, ARFGEF3, BCLAF1, CCDC28A, CITED2, ECT2L,*	3,847,985 bp	Uncertain significance	DECIPHER: (patient no. 524555)	NA	NA

	HEBP2, *HECA,* *IFNGR1,* *IL20RA,* *IL22RA2,* *MAP3K5,* *MAP7,* *MTFR2,* *NHSL1,* *OLIG3,* *PBOV1,* *PDE7B,* *PERP,* *PEX7,* *REPS1,* *SLC35D3,* *SMIM28,* *TNFAIP3*					
6q25 6:155364154 – 157681073 (GRCh37)	*ARID1B,* *NOX3,* *TMEM242,* *ZDHHC14*	2,316,919 bp	Uncertain significance	doi: 10.1016/j.ajhg.2012.02.007 (patient no. 1)	NA	NA

6q25 6:155966487 -158803979 (GRCh37)	*ARID1B, GTF2H5, SERAC1, SNX9, SYNJ2, TMEM242, TULP4, ZDHHC14*	2,837,492 bp	Pathogenic	doi: 10.1186/s12920-021-01119-2 (patient no. 1)	NA	NA
6q27 6:165220819 -170612142 (GRCh38)	*AFDN, C6orf118 C6orf120, CCR6, CEP43, DACT2, DLL1, DYNLT2, ERMARD, FAM120B, FRMD1, GPR31, KIF25, MPC1, PDCD2, PDE10A, PHF10, PRR18, PSMB1, RAMACL, RNASET2, RPS6KA2, SFT2D1, SMOC2, TBP, TBXT, THBS2, TTLL2,*	5,208,857 bp	Likely pathogenic	doi: 10.1002/ajmg.a.34079 (patient no. 2)	Sniffin' Sticks Test, MRI	Anosmia

	UNC93A, WDR27					
6q27 6:165126689 -170611942 ((GRCh38)	*AFDN, C6orf118* *C6orf120, CCR6, CEP43, DACT2, DLL1, DYNLT2, ERMARD, FAM120B, FRMD1, GPR31, KIF25, MPC1, PDCD2, PDE10A, PHF10, PRR18, PSMB1, RAMACL, RNASET2, RPS6KA2, SFT2D1, SMOC2, TBP, TBXT, THBS2, TTLL2, UNC93A, WDR27*	5,302,787 bp	Likely pathogenic	doi: 10.1002/ajmg.a.34079 (pateint no. 1)	MRI	NA
7p15.3 7:20356816-	*ITGB8*	93,530 bp	Uncertain significance	DECIPHER: (patient no. 389940)	Self-report	Anosmia

20452349 (GRCh38)						
7q21.11 7: 83833300-84046323 (GRCh38)	*SEMA3A*	213,024 bp	Likely pathogenic	ClinVar NC_000007.14:g.(83833300_?)_(?_84046323)del doi: 10.1093/humrep/des022	Self-report	Anosmia
7q21.11 7:83587561-83824314 (GRCh37)	*SEMA3A*	236,753 bp	Pathogenic	doi: 10.1210/clinem/dgac300 (patient no. 15)	UPSIT/self-report	Anosmia
8p11.22 8: 35401812-40555017 (GRCh37)	*ADAM18, ADAM2, ADAM32, ADAM9, ADRB3, AGPAT6, ANK1, AP3M2, ASH2L, BAG4, BRF2, C8orf4, C8orf86, DDHD2, EIF4EBP1, ERLIN2, FGFR1, GINS4, GOLGA7, GOT1L1, GPR124, HTRA4, IDO1, IDO2, KAT6A, LETM2, LSM1,*	5,153,205 bp	Pathogenic	doi: 10.1210/clinem/dgac300 (patient no. 7)	UPSIT/self-report	Anosmia

	NKX6-3, PLAT, PLEKHA2, PPAPDC1B, PROSC, RAB11FIP1, RP11-863K10.7, SFRP1, STAR, TACC1, TM2D2, WHSC1L1, ZMAT4, ZNF703					
8p11.22 8: 37553171-39142533 (GRCh37)	*AC012215.1, ADAM18, ADAM2, ADAM32, ADAM9, ADRB3, ASH2L, BAG4, BRF2, C8orf4, C8orf86, DDHD2, EIF4EBP1, ERLIN2, FGFR1, GOT1L1, GPR124, HTRA4, IDO1, IDO2, KCNU1, LETM2, LSM1,*	1,589,362 bp	Pathogenic	doi: 10.1210/clinem/dgac300 (patient no. 8)	UPSIT/self-report	Anosmia

	PLEKHA2, PPAPDC1B, PROSC, RAB11FIP1, RP11-863K10.7, STAR, TACC1, TM2D2, UNC5D, WHSC1L1, ZMAT4, ZNF703					
8p11.22 8: 37553171-42050826 (GRCh37)	*ADAM32, ADAM9, ADRB3, ASH2L, BAG4, BRF2, C8orf86, DDHD2, EIF4EBP1, ERLIN2, FGFR1, GOT1L1, GPR124, HTRA4, LETM2, LSM1, PLEKHA2, PPAPDC1B, PROSC, RAB11FIP1, RP11-863K10.7, STAR, TACC1,*	4,497,655 bp	Pathogenic	doi: 10.1210/clinem/dgac300 (patient no. 9)	UPSIT/self-report	Anosmia

	TM2D2, WHSC1L1, ZNF703					
8q12.2 8:59755307-64522596 (GRCh38)	*CHD7, ASPH, CA8, CLVS1, GGH, NKAIN3, RAB2A, TTPA, YTHDF3*	4,767,289 bp	Uncertain significance	DECIPHER: (patient no. 270126)	NA	NA
8q12.2 8:57854856-62246134 (GRCh38)	*CHD7, ASPH, CA8, CLVS1, CYP7A1, FAM110B, NSMAF, RAB2A, SDCBP, TOX, UBXN2B*	4,391,278 bp	Pathogenic	DECIPHER: (patient no. 472516)	NA	NA
8q12.2	*CHD7*	2,300,000 bp	Uncertain significance	doi: 10.1038/ng1407	NA	NA
9q31.2 9:108331353-110707332 (GRCh37)	*FKTN, ZNF462, TAL2, TMEM38B, RAD23B, KLF4*	2,375,979 bp	Uncertain significance	doi: 10.1530/EJE-20-1387	Self-report, MRI	Anosmia
12p11.2 12:27003224–31687824 (GRCh38)	*INTS13, TM7SF3, STK38L, ARNTL2, PPFIBP1,*	4,684,600 bp	Uncertain significance	doi: 10.1038/s41598-023-40037-4 (patient no. 1: DGAP032)	Self-report and olfactory test	Anosmia

	REP15, FAR2, ERGIC2, TMTC1, TSPAN11, DENND5B, ETFBKMT					
13q12.12 13: 22979193-24336627 (GRCh38)	*C1QTNF9, C1QTNF9B, MIPEP, SACS, SGCG, SPATA13, TNFRSF19*	1,357,434 bp	Likely pathogenic	DECIPHER: (patient no. 501263)	Self-report	Anosmia
15q24.1	*BBS4*	~6,000 bp	Pathogenic	doi: 10.1002/ajmg.a.30512 (patients 1)	UPSIT and CC-SIT	Anosmia
15q24.1	*BBS4*	~6,000 bp	Pathogenic	doi: 10.1002/ajmg.a.30512 (patients 3)	UPSIT and CC-SIT	Hyposmia
20q13.33 20:63166169 - 63697588(GRCh38)	*ARFGAP1, BIRC7, CHRNA4, COL20A1, EEF1A2, FNDC11, GMEB2, HELZ2, KCNQ2, NKAIN4, PPDPF, PTK6, RTEL1, RTEL1-*	531,419 bp	Likely pathogenic	DECIPHER: (patient no. 407019)	Self-report	Anosmia

	TNFRSF6B, SRMS, STMN3, TNFRSF6B, YTHDF1					
22q11.2 22:20731986 -21465672 (GRCh37)	*AIFM3, ARVCF, C22orf39, CCDC188, CDC45, CLDN5, CLTCL1, COMT, CRKL, DGCR2, DGCR6L, DGCR8, ESS2, FAM246C, GNB1L, GP1BB, GSC2, HIRA, KLHL22, LRRC74B, LZTR1, MED15, MRPL40, P2RX6, PI4KA, PRODH, RANBP1, RTL10, RTN4R,*	1,500,000-2,540,000 bp	Pathogenic	doi: 10.1542/peds.2005-3114	UPSIT	Anosmia

	SCARF2, SEPTIN5, SERPIND1, SLC25A1, SLC7A4, SNAP29, TANGO2, TBX1, THAP7, TRMT2A, TSSK2, TUBA3FP, TXNRD2, UFD1, ZDHHC8, ZNF74					
Xp22.31 X: 8531912-8535179 (GRCh38)	*ANOS1*	3,268 bp	Pathogenic	ClinVar NM_000216.4(ANOS1):c.1842+412_*1083del doi: 10.1056/NEJM199206253262606	Smell Identification Test	Anosmia
Xp22.3 X:5976388-8791727 (GRCh38)	*ANOS1, FAM9A, NLGN4X, PNPLA4, PUDP, STS, VCX, VCX2, VCX3A, VCX3B*	2,815,339 bp	Uncertain significance	DECIPHER: (patient no. 1740)	Self-report	Anosmia
Xp22.3 X:6938911-	*ANOS1, STS*	1,596,237 bp	Likely pathogenic	doi: 10.1111/and.12397	Olfactory tests	Anosmia

8535148 (GRCh38)		EX9-14 DEL				
Xp22.3 X:8536480-8730416	*ANOS1, FAM9A*	193,936 bp	Pathogenic	doi: 10.1111/and.12961	MRI	Anosmia
Xp22.3	*ANOS1*	EX3-13 DEL	Pathogenic	doi: 10.1210/jc.2002-021981	Olfactometr	Anosmia
Xp22.3	*ANOS1*	EX5-10DEL	Pathogenic	doi:10.1590/S1415-47572004000300006	Self-report	Anosmia
Xp22.3	*ANOS1, STS*	EX1-3DEL	Pathogenic	doi:10.1046/j.1365-2265.1998.00406.x	Olfactory testing (PEMEC)	Anosmia
Xp22.3	*ANOS1*	EX7	Likely pathogenic	doi: 10.1530/EC-22-0413 (patient no. 1)	Self-report	Anosmia
Xp22.3 X: 2771923-9734102 (GRCh37)	*ANOS1, ARSD, ARSF, ARSH, ARSL, FAM9A, FAM9B, GPR143, GYG2, MXRA5, NLGN4X, PNPLA4, PRKX, PUDP, STS,*	6,962,179 bp	Pathogenic	doi: 10.1210/clinem/dgac300 (patient no. 4)	UPSIT/self-report	Anosmia

	TBL1X, VCX, VCX2, VCX3A, VCX3B					
Xp22.3 X: 2771923-8536514 (GRCh37)	*ANOS1, ARSD, ARSF, ARSH, ARSL, GYG2, MXRA5, NLGN4X, PNPLA4, PRKX, PUDP, STS, TBL1X, VCX, VCX2, VCX3A, VCX3B*	5,764,591 bp	Pathogenic	doi: 10.1210/clinem/dgac300 (patient no. 5)	UPSIT/self-report	Anosmia
Xp22.3 X: 6966863-8591808 (GRCh37)	*ANOS1, PNPLA4, PUDP, STS, VCX, VCX2, VCX3A,*	1,624,945 bp	Pathogenic	doi: 10.1210/clinem/dgac300 (patient no. 1)	UPSIT/self-report	Anosmia
Xp22.3 X:7137374-8700325 (GRCh37)	*ANOS1, PNPLA4, STS, VCX, VCX2, VCX3B*	1,562,951 bp	Pathogenic	doi: 10.1210/clinem/dgac300 (patient no. 3)	UPSIT/self-report	Anosmia
Xp22.3 X:6966863-8700325 (GRCh37)	*ANOS1, PNPLA4, PUDP, STS, VCX, VCX2, VCX3B*	1,733,462 bp	Pathogenic	doi: 10.1210/clinem/dgac300 (patient no. 2)	UPSIT/self-report	Anosmia

Xp22.3 X:8521895-8591808	*ANOS1*	69,913 bp	Pathogenic	doi: 10.1210/clinem/dgac300 (patient no. 17)	UPSIT/self-report	Anosmia
Xp22.3 X:8521895-8700325	*ANOS1*	178,430 bp	Pathogenic	doi: 10.1210/clinem/dgac300 (patient no. 18)	UPSIT/self-report	Anosmia
Xp22.3 X:8538442-8700325	*ANOS1*	161,883 bp	Pathogenic	doi: 10.1210/clinem/dgac300 (patient no. 19)	UPSIT/self-report	Anosmia
Xp22.3	*STS, ANOS1*	NA	Pathogenic	doi: 10.1111/j.1365-2265.1995.tb01851.x (patients no. 1,2 and 3)	Olfactory test	Anosmia
Xp22.3	*STS, ANOS1, MRX*	~3,500,000 bp	Likely pathogenic	doi: 10.1111/j.1399-0004.1998.tb03692.x	Semiquantitative olfactory and trigeminal testing	Anosmia
Xp22.3 X:5810838-9733877	*STS, ANOS1, GPR143, NLGN4X, VCX-A, PUDP, PNPLA4*	3,923,039 bp	Likely pathogenic	doi: 10.3389/fgene.2020.00596 (patient no. 1)	MRI	Anosmia
Xp22.3	*ANOS1,*<br>*STS,*<br>*VCX-A,*<br>*OA1*	NA	Likely pathogenic	doi: 10.1210/jc.2003-030476 (patients no. 8, 9, 10 and 11)	MRI and olfactory testing (Toyota–Takagi/Alinamin)	Anosmia
Xp22.3	*STS, ANOS1, NLGN4X, VCX-3A, VCX-*	~3,700,000 bp	Likely pathogenic	doi: 10.1016/j.ejmg.2007.11.002	Olfactory test	Anosmia

	1, VCX-2, VCX-3B					
Xp22.3	*ANOS1, FAM9A, FAM9B, VCX-2r, VCX3*	~1,100,000 bp	Likely pathogenic	doi: 10.1159/000114858 (patients A1 and A2)	NR	Anosmia
Xp22.3 X:8536480 - 8730416 (GRCh37)	*ANOS1*	193,936 bp	Pathogenic	doi: 10.1111/and.12961.	NR	Anosmia
Xp22.3	*ANOS1*	~3,500,000 bp	Pathogenic	doi: 10.1111/j.1399-0004.1998.tb03692.x	Qualitative and semiquantitative olfactory/trigeminal testing	Anosmia

Supplementary Table S3. Characteristics of identified microduplications, including chromosomal location, protein-coding gene content, variant size, clinical classification, method of olfactory dysfunction (OD) assessment or method not reported (NR), and olfactory phenotype (anosmia, hyperosmia, or not assessed (NA)).

Chr. region	Protein-coding genes	Length	Classification	Source/database	Diagnosis of OD	Olfactory phenotype
2q14.3 2: 128262169-	*HS6ST1*	12,887 bp	Uncertain significance	ClinVar	NA	NA

128275055 (GRCh38)				NC_000002.12:g.128262169_128275055dup		
2q21.1 2:130724411-131193847 (GRCh38)	*AMER3, ARHGEF4, FAM168B, GPR148, PLEKHB2, SMIM39*	469,437 bp	Uncertain significance	DECIPHER: (patient no. 255882)	NR	Anosmia
4q24 4:100985336-104664827 (GRCh38)	*BANK1, BDH2, CENPE, CISD2, CXXC4, MANBA, NFKB1, PPP3CA, SLC39A8, SLC9B1, SLC9B2, TACR3, UBE2D3*	3,679,491 bp	Uncertain significance	DECIPHER: (patient no. 383443)	NA	NA

6q23.3 6: 137143759-137167339 (GRCh37)	*PEX7*	23,581 bp	Uncertain significance	ClinVar NC_000006.11:g.(?_137143759)_(137167339_?)dup	NA	NA
6q23.3 6: 137187765-137191141 (GRCh37)	*PEX7*	3,377 bp	Likely pathogenic	ClinVar NC_000006.11:g.(?_137187765)_(137191141_?)dup	NA	NA
6q23.3 6: 137142702-137236072 (GRCh37)	*PEX7, RPL7AP37*	93,371 bp	Uncertain significance	ClinVar NC_000006.12:g.(?_136821564)_(136914934_?)dup	NA	NA
8q12.2 8:59307187-63159480 (GRCh38)	*ASPH, CA8, CHD7, CLVS1, GGH, NKAIN3, RAB2A, TTPA*	3,852,293 bp	Uncertain significance	DECIPHER: (patient no. 267338)	NA	NA
8q12.2 8:60508904-	*CHD7, CLVS1, RAB2A*	613,643 bp	Uncertain significance	DECIPHER: (patient no. 384447)	NA	NA

61122547 (GRCh38)						
8q12.2 8: 58388614-65306097 (GRCh37)	*ASPH, CA8, CHD7, CLVS1, CYP7A1, FAM110B, GGH, NKAIN3, NSMAF, RAB2A, SDCBP, TOX, TTPA, UBXN2B, YTHDF3*	6,917,483 bp	Uncertain significance	doi: 10.1016/j.ejmg.2009.09.006	NA	NA
20p13 20:388596-480675 (GRCh37)	*CSNK2A1, RBCK1, TBC1D20*	92,079 bp	Uncertain significance	doi: 10.1210/clinem/dgac300 (patient no. 24)	UPSIT/self-report	Anosmia

Xp22.31	*STS, ANOS1, DXS1283E, VCX3B, HDHD1A*	1,290,000 bp - 2,350,000 bp	Benign/Likely benign	GEO: GSE299701<br>doi: 10.1186/s12884-026-09837-0	NA	NA
Xp22.31 X:8596840–8705155 (GRCh37)	*ANOS1*	108,315 bp	Pathogenic	doi: 10.1007/s13353-014-0252-7	Quantitative olfactory examination	Hyperosmia
Xp22.31 X:8553210-8591808 (GRCh38)	*ANOS1*	38,598 bp	Pathogenic	doi: 10.1210/clinem/dgac300 (patient no. 26)	UPSIT/self-report	Anosmia
Xp22.31 X:8521895-8591808 (GRCh38)	*ANOS1*	69,913 bp	Pathogenic	doi: 10.1210/clinem/dgac300 (patient no. 28)	UPSIT/self-report	Anosmia
